# Monocyte Subsets and Related Chemokines in Carotid Artery Stenosis and Ischemic Stroke

**DOI:** 10.3390/ijms17040433

**Published:** 2016-03-23

**Authors:** Gerrit M. Grosse, Walter J. Schulz-Schaeffer, Omke E. Teebken, Ramona Schuppner, Meike Dirks, Hans Worthmann, Ralf Lichtinghagen, Gerrit Maye, Florian P. Limbourg, Karin Weissenborn

**Affiliations:** 1 Department of Neurology, Hannover Medical School, 30625 Hannover, Germany; grosse.gerrit@mh-hannover.de (G.M.G.); schuppner.ramona@mh-hannover.de (R.S.); dirks.meike@mh-hannover.de (M.D.); worthmann.hans@mh-hannover.de (H.W.); 2 Department of Neuropathology, University Medical Center Göttingen, 37099 Göttingen, Germany; wjschulz@med.uni-goettingen.de; 3 Department of Vascular Surgery, Klinikum Peine, 31226 Peine, Germany; omke.teebken@klinikum-peine.de; 4 Department of Clinical Chemistry, Hannover Medical School, 30625 Hannover, Germany; lichtinghagen.ralf@mh-hannover.de; 5 Department of Nephrology and Hypertension, Hannover Medical School, 30625 Hannover, Germany; gerritmaye@gmail.com (G.M.); limbourg.florian@mh-hannover.de (F.P.L.); 6 Center for Systems Neuroscience (ZSN), 30559 Hannover, Germany

**Keywords:** atherosclerosis, biomarkers, carotid stenosis, ischemic stroke, plaque analysis

## Abstract

Carotid stenosis (CS) is an important cause of ischemic stroke. However, reliable markers for the purpose of identification of high-risk, so-called vulnerable carotid plaques, are still lacking. Monocyte subsets are crucial players in atherosclerosis and might also contribute to plaque rupture. In this study we, therefore, aimed to investigate the potential role of monocyte subsets and associated chemokines as clinical biomarkers for vulnerability of CS. Patients with symptomatic and asymptomatic CS (*n* = 21), patients with cardioembolic ischemic strokes (*n* = 11), and controls without any cardiovascular disorder (*n* = 11) were examined. Cardiovascular risk was quantified using the Essen Stroke Risk Score (ESRS). Monocyte subsets in peripheral blood were measured by quantitative flow cytometry. Plaque specimens were histologically analyzed. Furthermore, plasma levels of monocyte chemotactic protein 1 (MCP-1) and fractalkine were measured. Intermediate monocytes (Mon2) were significantly elevated in symptomatic and asymptomatic CS-patients compared to controls. Mon2 counts positively correlated with the ESRS. Moreover, stroke patients showed an elevation of Mon2 compared to controls, independent of the ESRS. MCP-1 levels were significantly higher in patients with symptomatic than in those with asymptomatic CS. Several histological criteria significantly differed between symptomatic and asymptomatic plaques. However, there was no association of monocyte subsets or chemokines with histological features of plaque vulnerability. Due to the multifactorial influence on monocyte subsets, the usability as clinical markers for plaque vulnerability seems to be limited. However, monocyte subsets may be critically involved in the pathology of CS.

## 1. Introduction

Carotid artery stenosis (CS) is a common manifestation of atherosclerosis [1] and an important cause of ischemic stroke with 10%–15% being caused by CS [2]. With carotid endarterectomy (CEA) and percutaneous carotid stenting there are effective secondary preventive therapies available for well-selected patients. Though, for patients with asymptomatic high-grade (*i.e.*, ≥70%) CS indication for CEA is challenging, since the probable overall benefit is relatively low with an absolute risk reduction of 2%–3% concerning a three-year follow up [3]. Even equivalence of CEA and best medical treatment has been proposed in managing asymptomatic CS [4]. Therefore, identification of patients with high-risk, so-called vulnerable CS, is highly warranted for the purpose of risk stratification and for an appropriate indication for therapy, since grade of stenosis alone might be a misleading feature.

Monocytes, as precursors of macrophages, are known to be crucial players in the process of atherosclerosis. Monocytes consist of subsets which can be differentiated according to their expression of the lipopolysaccharide (LPS) receptor CD14 and the FcγIII receptor CD16 [5]. Classical monocytes (CD14^++^CD16^−^; Mon1) constitute the major subpopulation with about 85%, the intermediate subset (CD14^++^CD16^+^; Mon2) make up about 5%, and nonclassical monocytes (CD14^+^CD16^++^; Mon3) about 10% [6,7]. Monocyte subsets seem to adopt diverse functions in different (mainly inflammatory) pathologies, although the exact functions are still controversial. The invasion of monocyte subsets into tissue depends, besides others, on the chemokines monocyte chemotactic protein 1 (MCP-1; CCL2) and fractalkine (FKN; CX3CL1). The corresponding receptors are presented on monocytes with CCR2 being mainly expressed on Mon1 and Mon2, and CX3CR1 mainly on Mon2 and Mon3 [8,9].

Recently, a predictive pattern of monocyte subsets has been proposed for cardiovascular events [10,11]. With its specific receptor profile, especially Mon2 seem to carry out pro-atherosclerotic functions [12,13]. Moreover, an association of computer tomography (CT) detected vulnerable atherosclerotic plaques with CD16+ monocytes was reported in patients with coronary heart disease (CHD) [14]. Therefore, monocyte subsets could also serve as promising biomarkers in CS. In this pilot study we, therefore, aimed to investigate the usability of monocyte subsets and associated chemokines as biomarkers for vulnerability in human CS.

## 2. Results

### 2.1. Study Population

For epidemiological data see Table 1. According to the study design, there were significant differences between the study groups regarding the ESRS (*p* = 0.008), with Co showing significantly lower ESRS levels than sCS and aCS (*p* = 0.026, *p* = 0.009, respectively, according to *post hoc* analysis). Other baseline characteristics did not significantly differ.

### 2.2. Monocyte Subset Counts

**Figure 1:**
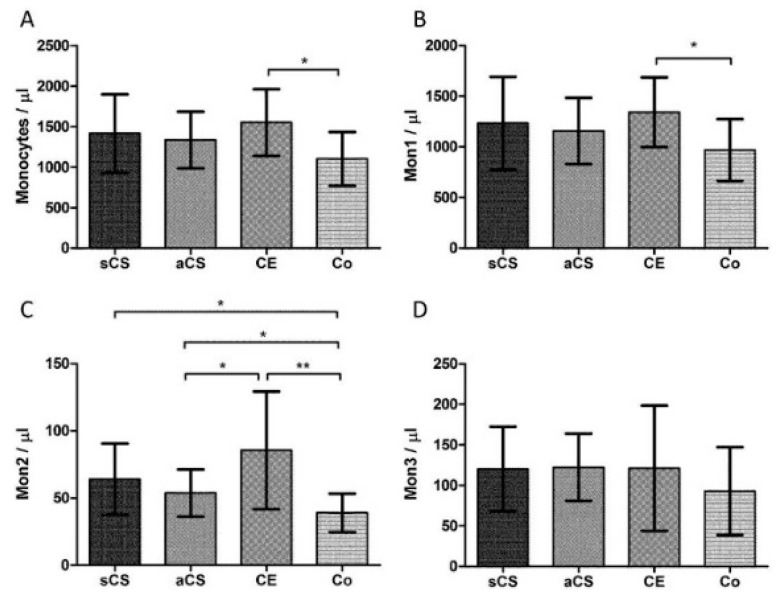
Monocyte subset counts (means ± SD). (**A**) Total monocyte counts; (**B**) counts of classical monocytes (Mon1); (**C**) counts of intermediate monocytes (Mon2); and (**D**) counts of non-classical monocytes (Mon3). aCS: asymptomatic carotid artery stenosis; CE: cardioembolic stroke; Co: controls; sCS: symptomatic carotid artery stenosis. * *p* < 0.05 ** *p* < 0.01.

Absolute counts of Mon2 showed significant differences with higher counts in sCS *vs.* Co (*p* = 0.015), aCS *vs.* Co (*p* = 0.049), and CE *vs.* Co (*p* = 0.006) (Figure 1C). For Mon1 and total monocytes differences were observed between CE and Co (*p* = 0.014 and *p* = 0.011, respectively) (Figure 1A,B). There were no differences of monocyte counts between sCS and aCS. Additionally, for Mon3, no differences could be found within the study population (Figure 1D).

### 2.3. Monocyte Subsets after Ischemic Stroke

In a pooled stroke patient analysis (sCS + CE) differences of Mon2 counts could be underlined: With a factor of 1.9 stroke patients showed significantly higher counts of Mon2 compared to controls (*p* < 0.001), in mean 5 days after stroke. Higher counts could be as well observed for total monocyte counts (*p* = 0.017) and Mon1 (*p* = 0.027). A multivariate logistic regression analysis including ESRS revealed that elevated Mon2 counts were independently present in stroke patients (*p* = 0.03).

Concerning stroke severity, we compared mild strokes (NIHSS 0-1) with moderate/severe strokes (NHSS > 1). Relative, but not absolute, monocyte subset levels showed differences only by tendency: he proportion of Mon1 was higher in patients with moderate/severe strokes (*p* = 0.069), whereas the proportion of Mon3 was higher in patients with mild strokes (*p* = 0.055).

### 2.4. Monocyte Subsets and Cardiovascular Risk

A potential association of monocyte subsets with cardiovascular risk was analyzed using data of patients with aCS and controls, exclusively. Thereby we found a positive correlation of Mon2 counts with the ESRS (*r* = 0.556; *p* = 0.009). Furthermore, Mon2 counts were associated with creatinine concentrations (*r* = 0.536; *p* = 0.018). Total monocyte counts and Mon1 counts positively correlated with hs-CRP (*r* = 0.597; *p* = 0.007 and *r* = 0.587; *p* = 0.008, respectively).

### 2.5. Levels of FKN and MCP-1

**Figure 2:**
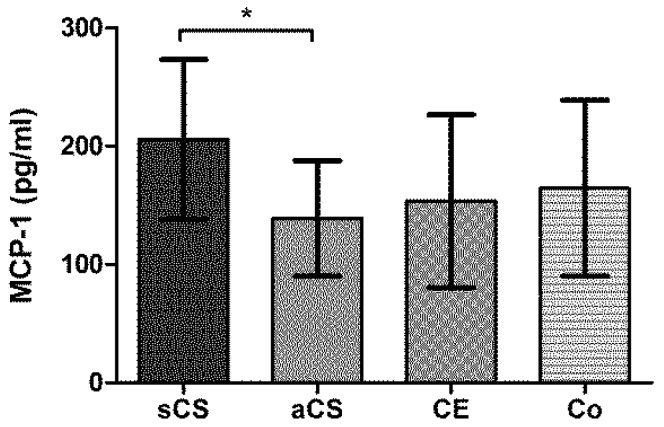
Plasma levels of MCP-1 (pg/mL). aCS: asymptomatic carotid artery stenosis; CE: cardioembolic stroke; Co: controls. sCS: symptomatic carotid artery stenosis. * *p* < 0.05.

FKN levels did not show any differences between the study groups, but we found an association with stroke severity, showing that FKN levels were significantly higher in patients with mild strokes compared to those with moderate/severe strokes (*p* = 0.003). MCP-1 levels were significantly higher in patients with sCS compared with patients with aCS (*p* = 0.033) (Figure 2).

### 2.6. Histological Analysis

By comparing the histological features of symptomatic *vs.* asymptomatic carotid artery stenosis, the symptomatic plaques presented a smaller area of vessel wall fibrosis (*p* = 0.003), a greater area of hemorrhage resorption (*p* = 0.005), and more cases with cap infiltration by mainly macrophages (*p* = 0.012; Table 2). “Active” plaques rated by plaque rupture and cap infiltration were more frequent in symptomatic carotid artery stenosis (*p* = 0.046). By tendency, macrophages (*p* = 0.083) and cholesterol crystals (*p* = 0.055) within the plaques were also more frequent in symptomatic carotid plaques (Table 2). Since stroke *per se* affects monocyte subset counts, associations between monocyte subset counts and histological plaque features were analyzed for patients with asymptomatic carotid artery stenosis exclusively, but did not show any correlation.

## 3. Discussion

In this pilot study we investigated, for the first time, absolute levels of monocyte subset counts and associated chemokines in peripheral venous blood in regard to histological features of vulnerability in human CS. We observed that Mon2 counts are significantly higher in patients with high-grade symptomatic, as well as asymptomatic CS compared to controls. This result resembles those of different studies regarding monocyte subsets in CHD in which a role of CD16+ monocytes (Mon2 and Mon3) has been proposed [14,15,16]. Furthermore, especially Mon2 seem to carry pro-atherogenetic and pro-inflammatory functions, probably due to the specific receptor profile and the generation of reactive oxygen species [12,13]. Thus, elevation of Mon2 counts in macroangiopathy could be cause of the vascular lesions instead of the consequence. Concerning cardiovascular risk, we found a positive correlation of Mon2 counts with the ESRS in aCS patients and controls, as well as with creatinine levels. Hence, this study supports results of other papers in which a predictive value of Mon2 counts for cardiovascular events has been outlined [10,17].

The histological analysis in this study led to a discriminability of sCS and aCS based on several features of inflammation and vulnerability. Monocyte subsets may contribute to plaque vulnerability and a linkage to rupture-prone plaques has been described in previous clinical studies for CHD [14,18]. Contrary to our hypothesis, we did not find a difference of monocyte subset counts, neither between sCS and aCS, nor in regard to histological criteria of vulnerability. However, we observed that predominantly Mon2 counts differed between acute stroke patients and controls. The multivariate analysis showed that elevation of Mon2 counts in stroke patients is independent from cardiovascular risk, as measured using the ESRS. Thus, the comparison of sCS with aCS may be hampered by the acute event and following post-ischemic inflammatory processes. We, therefore, analyzed monocyte subset counts within the aCS-group in regard to histological characteristics of carotid plaques but, again, did not find any differences between monocyte subset counts, probably due to the relatively small number of cases.

Jaipersad *et al.*, recently published data of absolute monocyte subset counts in patients with CS according to ultrasound examination [19]. Interestingly, the authors describe an association of Mon1, but not of Mon2, with presence and degree of CS. This discrepancy might be due to different gating strategies for measuring monocyte subsets: Jaipersad *et al.*, distinguished Mon2 and Mon3 by expression of CCR2, while in this study expression of CD16 has been used for discrimination of those two subsets. Jaipersad *et al.*, moreover found a linkage of Mon1 with neovascularization within carotid plaques which was detected by ultrasound. Neovascularization contributes to plaque rupture and is a frequent histological feature in CS. Additionally, in all specimens in our study, neovessels could be found. These data further support an association of monocyte subsets with the pathology of CS. The different results of the two studies concerning Mon2 and Mon3 counts may demonstrate that consistent gating strategies for FACS-analysis would be beneficial for future studies.

As outlined above, Mon2 counts are also significantly elevated in acute stroke patients compared to controls. This is in line with the time course of monocyte subset proportions in ischemic stroke patients that demonstrated increased proportions of Mon1 and Mon2 after the event [20]. In our current study, the proportions of Mon1 and Mon3 tended to differ depending on stroke severity. In concordance to our findings, Urra *et al.* [21] reported an association of the proportions of Mon3 with favorable outcome and of Mon1 with unfavorable outcome after ischemic stroke. Interestingly, we did not find differences of absolute monocyte subset counts in regard to stroke severity but only for the subset ratios. In previous studies, absolute monocyte subset counts have not been investigated in stroke patients. A possible role of monocyte subsets in post-stroke inflammation has been discussed previously based on animal experiment data [22]. However, it remains controversial if there is an active influence in the pathophysiology of stroke since a recent study proposes that Ly6C^low^ monocytes are redundant for outcome in an experimental murine stroke model [23]. Murine Ly6C^low^ monocytes are regarded as being comparable to human nonclassical monocytes. However, further investigation is warranted to elucidate the role of monocyte subsets in human ischemic stroke.

MCP-1 and FKN and its corresponding receptors are critically involved in the recruitment of monocyte subsets into tissue [24,25]. We found a significant difference of MCP-1 levels between sCS and aCS, with symptomatic patients showing higher levels. Other differences were not observed within the study population. As reported previously, MCP-1 levels are also elevated in acute stroke with a rapid regulation within 12 h [26]. A possible influence on the current results cannot be completely ruled out, but seems to be unlikely since blood was drawn after the described peak time window. We failed to confirm a possible association of MCP-1 levels with histological evaluated plaque vulnerability. Nevertheless, MCP-1 should be further investigated as a potential biomarker in CS since an association with plaque vulnerability has already been described in experimental studies [27,28].

Surprisingly, there were no differences of FKN levels within the study population, although it has been regarded as pro-atherosclerotic [24,29]. This may be due to the fact that mainly patients with Parkinson’s disease (PD) served as controls. They were chosen because of the low incidence of cardiovascular morbidities in this patient group. On the other hand, there are recent hints of FKN playing also a role in PD, although this has not been confirmed in patients, yet [30,31]. Instead, FKN levels showed remarkable results regarding stroke severity consistent with previous data from our group [32]. There has been a recent report by Grozdanov *et al.* [33], describing an alteration of relative monocyte subset counts in patients suffering from PD, like most of the controls in this study. Importantly, many of those PD patients additionally had at least one cardiovascular risk factor in the study of Grozdanov *et al.* [33]. Thus, the disturbance of monocyte subset constitution might be as well due to presence of risk factors and less as a result of PD, as supported by the current results and those lined out above. At least, the hypothetic role of monocyte subsets as a systemic marker in neurodegenerative disorders needs to be further investigated.

There are some limitations of this study. First of all, the patient cohort is relatively small, due to the proper selection and diverse investigations, and our findings need to be proven in future studies with a larger cohort. Moreover, histological analysis and chemokine measurements could not be performed for all patients. Furthermore, it cannot be completely ruled out that, due to the intraoperative preparation, parts of carotid plaques may have not been considered. As shown previously, monocyte subsets are involved and altered in different pathologies. Thus, despite of strict exclusion criteria, it cannot be ruled out that there are even yet unrecognized influencing variables which may affect monocyte subsets within this study. As a result, monocyte subsets might have too low a specificity for the purpose as clinical biomarkers. Though, in view of latest research, it is very likely that monocyte subsets play an intervening role in the initiation and progression of CS.

## 4. Material and Methods

### 4.1. Study Population

Eleven patients with symptomatic carotid artery stenosis (sCS), ten patients with asymptomatic carotid artery stenosis (aCS), eleven patients with cardioembolic ischemic strokes (CE), and eleven age- and gender-matched controls without cardiovascular disorders (Co) were included in this study. Ten of the control patients suffered from Parkinson’s disease (PD), one was a healthy subject. Subjects were recruited between January 2011 and June 2012 at the Department of Neurology (patients with sCS) and the Department of Cardiothoracic, Transplantation, and Vascular Surgery (patients with aCS) at Hannover Medical School. Exclusion criteria were acute infections, current malignant disease, cerebral hemorrhage, and immunosuppressive medication. In patients with cardioembolic strokes, stenosis of the brain supplying arteries was ruled out using Doppler ultrasound, and either computer tomographic or magnetic resonance angiography. All controls underwent Doppler ultrasound for exclusion of stenosis of the brain supplying arteries. Blood was drawn after stroke etiology was determined and before surgery with a maximum of 18 h before CEA in groups sCS and aCS.

The study was approved by the local ethics committee. All patients or legal representatives and volunteers provided written informed consent before inclusion into the study.

### 4.2. Clinical Evaluation

Demographical and clinical data including cardiovascular risk factors (CVRF) were obtained for patients and controls. The cardiovascular risk was graduated using the Essen Stroke Risk Score (ESRS) (comprising the factors age, arterial hypertension, diabetes mellitus, previous myocardial infarction, other cardiovascular disease, peripheral arterial disease, nicotine consumption, previous stroke or transient ischemic attack) [34]. Stroke severity was scored using the National Institutes of Health Stroke Scale (NIHSS) on admission. Groups were defined as mild (NIHSS of 0–1) and moderate/severe strokes (NIHSS > 1) [35]. All subjects underwent Doppler-ultrasound of the brain-supplying arteries. In addition, all stroke patients underwent *trans*-thoracic/-esophageal echocardiography and imaging studies of the brain and brain-supplying arteries (cranial computer tomography or magnetic resonance imaging). For the groups sCS and aCS, a three-month follow up examination was performed, including neurological examination and blood tests.

### 4.3. Flow Cytometry

**Figure 3:**
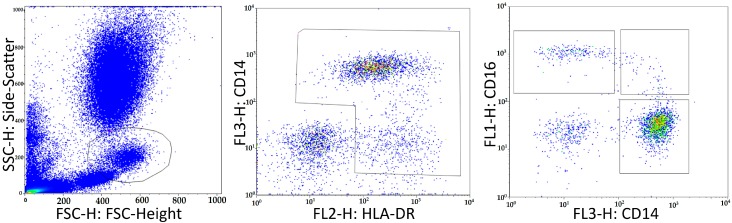
Gating strategy for FACS-analysis of monocyte subset counts. First, total monocytes were gated in a FSC/SSC-plot; Afterwards, in a CD14/HLA-DR-Plot HLA+ cells were gated for exclusion of lymphocytes; Finally, the three monocyte subsets could be measured in a CD16/CD14-plot.

Monocyte subsets were measured in whole blood samples using quantitative fluorescence-activated cell sorting (FACS)–analysis (FACScalibur, Becton, Dickinson and Company, Franklin Lakes, NJ, USA) with following antibodies: CD14 PerCP (clone MΦP9, Becton, Dickinson and Company), CD16 FITC (clone 3G8, Biolegend, San Diego, CA, USA), HLA-DR PE (clone L243, Biolegend). 100 µL of whole blood anticoagulated with EDTA were mixed with 10 µL of each antibody in a TruCount™ tube (Becton, Dickinson and Company). After incubation for 15 min erythrocytes were lysed according to the recommendations of the manufacturer. After another 15 min incubation time FACS-analysis was performed using BD CellQuest™ Pro (Becton, Dickinson and Company). Gating of monocytes was done using FlowJo (TreeStar Inc, Ashland, OR, USA). The gating strategy was based on selection of total monocytes in the forward scatter (FSC)/side scatter (SSC)-plot. In a CD14/HLA-DR-Plot HLA-positive cells could be identified and gated to exclude lymphocytes. Finally, the three monocyte subsets could be measured in a CD16/CD14-plot (see Figure 3). Using the TruCount™-System allowed to measure absolute monocyte counts.

### 4.4. Enzyme-Linked Immunosorbent Assay (ELISA) and Clinical Chemistry

Levels of MCP-1 and FKN were determined in peripheral venous blood samples which have been centrifuged with 3000 rpm for 10 min and then stored at −80 °C until measurement. Measurements were performed using ELISA-Kits (R and D Systems, Inc., Minneapolis, MN, USA). For measuring MCP-1 there were eight samples available for group sCS, nine for aCS, and 10 each for CE and Co. FKN could be measured in nine samples in each sCS and aCS, in 11 samples of CE, and 10 of Co. Furthermore, complete blood cell counts, creatinine-levels, and high-sensitive C-reactive-protein (hs-CRP) levels were provided.

### 4.5. Histological Analysis

In 16 cases (eight aCS and eight sCS patients) plaque specimens which were obtained during carotid endarterectomy were analyzed histologically by a pathologist (WJ S-S) blinded for the clinical status of the patients. The plaques were collected in phosphate buffered saline (PBS) immediately after preparation. Twelve samples were formalin-fixed immediately after collection; four additional samples that were previously shock frozen were later carefully defrosted and fixed in formalin. After demineralization with 20% EDTA at pH 7.4 for five days, the surgical samples were cut into series of 3 mm slices and embedded in paraffin. From the paraffin block 3 µm slices were cut and stained with haematoxylin and eosin (H and E) and an Elastica-van Gieson (EvG) stain. Immunohistochemically, an antigen reaction with the pan macrophage antibody KiM1P (provided by the Pathology Department at the University Hospital in Kiel, Germany [36]) was performed.

**Figure 4:**
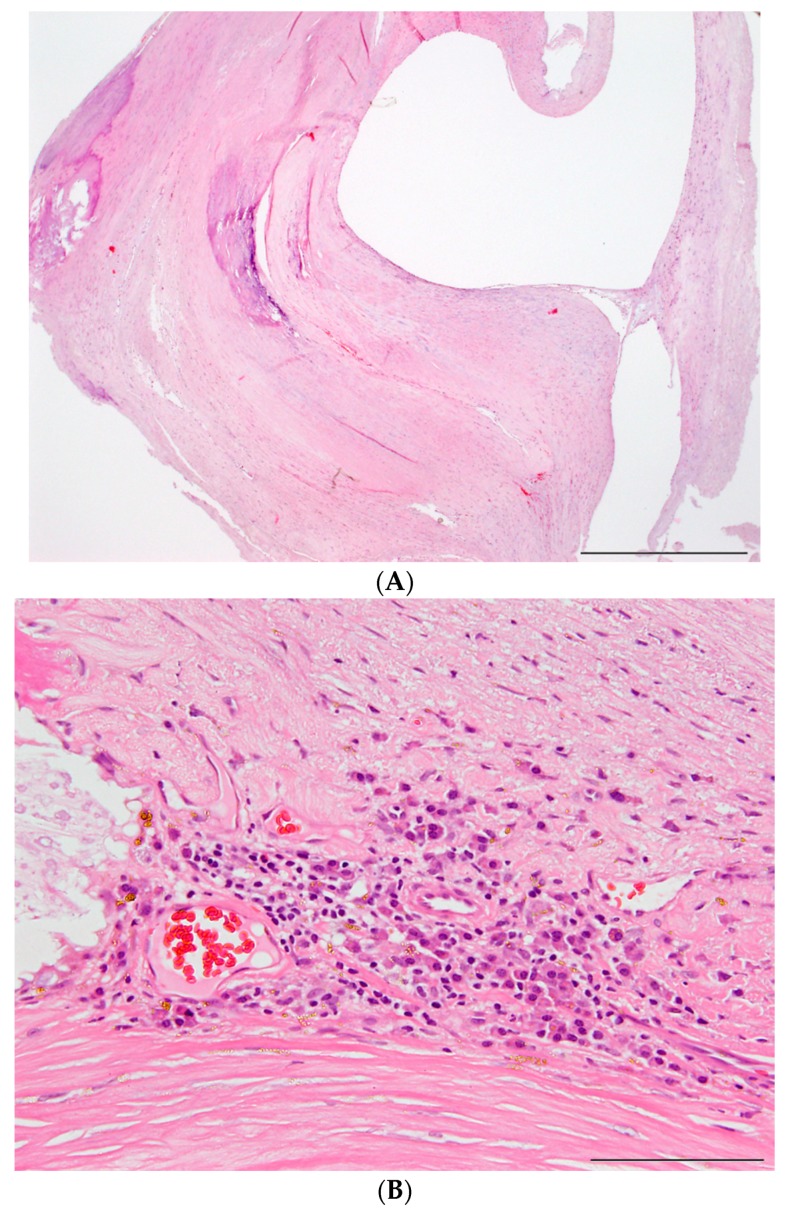

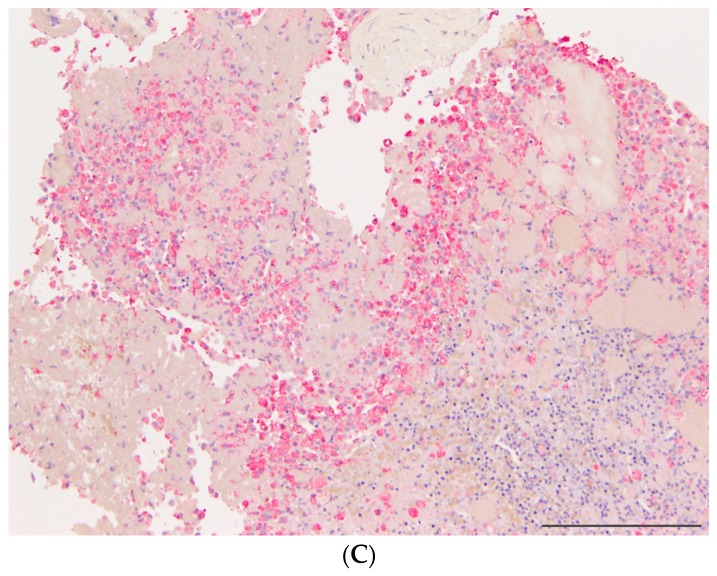
Examples of histological examined carotid plaques. (**A**) Example of a carotid plaque, stained H and E. The plaque is obliterating the lumen but shows mainly vessel wall fibrosis. No blood clot, no granulocytic or cap macrophage infiltration or plaque rupture is seen. The plaque has been regarded as “not active”. Bar size: 1 mm; (**B**) neovascularization and clustered lymphocyte infiltration at the border between carotid plaque and adventitia (H and E stain). Bar size: 100 µm; and (**C**) immunohistochemical staining of macrophages (red), infiltrating and resorbing a blood clot at the cap of an “active” carotid plaque. Monoclonal antibody KiM1P, visualized by neufuchsin. Bar size: 200 µm.

The slides were analyzed regarding following criteria: vessel wall fibrosis, resorption of hemorrhage, granulocytic and macrophage infiltration of hemorrhages and plaque rupture, presence of giant cells, vessel wall infiltration by lymphocytes and lymphocyte clusters, mineralization, neovascularization, and cholesterol crystals (for examples see Figure 4). Finally, by subjective evaluation of the pathologist and based mainly on presence or absence of plaque rupture and macrophage clusters decorating hemorrhage deposits on the surface to the blood stream (cap infiltration), the plaques were classified as “active” *vs.* “not active”.

### 4.6. Statistical Analysis

Statistical analysis was performed using IBM SPSS Statistics 20 (SPSS Inc., Chicago, IL, USA) and GraphPad Prism 5 (GraphPad Inc., La Jolla, CA, USA). Gaussian distribution was tested using the Kolmogorov–Smirnov test. Comparisons between groups were done using Student’s *t*-test for normally-distributed data or Man–Whitney *U*-test for non-normally distributed data and Chi-square test for categorical data. Group comparisons have been analyzed using ANOVA and *post hoc* analysis according to Bonferroni. Correlations were calculated with bivariate Pearson-correlation or Spearman correlation, as appropriate. A *p*-value <0.05 was regarded significant.

## 5. Conclusions

In this pilot study, we investigated the potential role of monocyte subsets and related chemokines as biomarkers for vulnerability of CS. Mon2 counts are altered both in ischemic stroke and under presence of cardiovascular risk factors. Our hypothesis of different monocyte subset counts representing plaque vulnerability in human CS could not be proven in this study. Their usability as clinical biomarkers might be limited due to low specificity. Nonetheless, monocyte subsets and related chemokines, like e.g., MCP-1, may be critically involved in the pathology of atherosclerosis and plaque vulnerability and should, therefore, be further investigated in larger studies.

## Figures and Tables

**Table 1:** Baseline characteristics.

	sCS	aCS	CE	Co	*p*-Value
*N*	11	10	11	11	
Age (a) ± SD	68.36 ± 8.99	74.80 ± 5.75	73.73 ± 14.66	65.36 ± 9.60	0.131
Sex	–	–	–	–	0.885
Male	9 (82%)	7 (70%)	9 (82%)	9 (82%)	–
Female	2 (18%)	3 (30%)	2 (18%)	2 (18%)	–
ESRS ± SD	2.82 ± 2.14	3.09 ± 0.54	2.40 ± 1.17	1.00 ± 0.82	0.008
BMI (kg/m^2^) ± SD	28.45 ± 4.78	27.74 ± 3.99	26.44 ± 4.75	26.20 ± 4.69	0.420
Dyslipidemia	5	5	4	0	0.055

The factors age, arterial hypertension, diabetes mellitus, previous myocardial infarction, other cardiovascular disease, peripheral arterial disease, nicotine consumption, previous stroke, or transient ischemic attack are subsumed in the ESRS. *p* < 0.05 is considered significant. a: years; aCS: asymptomatic carotid artery stenosis, BMI: body mass index; CE: cardioembolic stroke; Co: controls; ESRS: Essen stroke risk score; sCS: symptomatic carotid artery stenosis; SD: standard deviation.

**Table 2:** Histological analysis of CS.

Histological Feature	sCS (*n* = 8)	aCS (*n* = 8)	*p*-Value
vessel wall fibrosis (area) ± SD	39% ± 14.74	66% ± 14.82	0.003
macrophage infiltration (area) ± SD	4.25% ± 1.49	2.50% ± 1.77	0.083
haemorrhage resorption (area) ± SD	46% ± 15.62	16% ± 16.57	0.005
mineralization (area) ± SD	13% ± 12.50	18% ± 18.13	0.382
cap macrophage infiltration	75%	12.5%	0.012
plaque rupture	63%	25%	0.131
granulocyte infiltration	25%	0%	0.131
giant cells	38%	38%	1
lymphocyte clusters	75%	38%	0.131
neovascularisation	100%	100%	1
cholesterol crystals	100%	63%	0.055
active plaque	75%	25%	0.046

Histological features in carotid stenosis. aCS: asymptomatic carotid artery stenosis, sCS: symptomatic carotid artery stenosis. SD: standard deviation.
